# The p53-p21-DREAM-CDE/CHR pathway regulates G_2_/M cell cycle genes

**DOI:** 10.1093/nar/gkv927

**Published:** 2015-09-17

**Authors:** Martin Fischer, Marianne Quaas, Lydia Steiner, Kurt Engeland

**Affiliations:** 1 Molecular Oncology, Medical School, University of Leipzig, Leipzig, Germany; 2 Centre for Complexity & Collective Computation, Wisconsin Institute for Discovery, Madison, WI, USA; 3 Computational EvoDevo Group & Bioinformatics Group, Department of Computer Science, and Interdisciplinary Centre for Bioinformatics, University of Leipzig, Leipzig, Germany

## Abstract

The tumor suppressor p53 functions predominantly as a transcription factor by activating and downregulating gene expression, leading to cell cycle arrest or apoptosis. p53 was shown to indirectly repress transcription of the *CCNB2*, *KIF23* and *PLK4* cell cycle genes through the recently discovered p53-p21-DREAM-CDE/CHR pathway. However, it remained unclear whether this pathway is commonly used. Here, we identify genes regulated by p53 through this pathway in a genome-wide computational approach. The bioinformatic analysis is based on genome-wide DREAM complex binding data, p53-depedent mRNA expression data and a genome-wide definition of phylogenetically conserved CHR promoter elements. We find 210 target genes that are expected to be regulated by the p53-p21-DREAM-CDE/CHR pathway. The target gene list was verified by detailed analysis of p53-dependent repression of the cell cycle genes *B-MYB* (*MYBL2*), *BUB1*, *CCNA2*, *CCNB1*, *CHEK2*, *MELK*, *POLD1*, *RAD18* and *RAD54L*. Most of the 210 target genes are essential regulators of G_2_ phase and mitosis. Thus, downregulation of these genes through the p53-p21-DREAM-CDE/CHR pathway appears to be a principal mechanism for G_2_/M cell cycle arrest by p53.

## INTRODUCTION

The key functions of the tumor suppressor p53 are regulation of the cell cycle and induction of apoptosis. Its dominant role is to serve as a transcription factor by activating or downregulating expression of target genes (1,2). The gene of the cyclin-dependent kinase (CDK) inhibitor p21 (WAF1, CIP1, CDKN1A) was the first p53 transcriptional target to be identified and shown to be activated by p53 (3). Additionally, p21 was later implicated in mediating indirect transcriptional repression by p53 (4–12). A possible mechanism for indirect transcriptional repression is based on the cdk-inhibitor function of p21 which leads to hypophosphorylation of retinoblastoma tumor suppressor pRB-related pocket proteins. Low phosphorylation levels of the pRB-related p130 and p107 proteins then induce formation of a protein complex named DREAM (DP, RB-like, E2F4 and MuvB). DREAM assembles as a result of changing components of the MMB (MYB-MuvB) complex (13–15). In contrast to the activating MMB complex, DREAM is a transcriptional repressor (15–18). The p53-p21-DREAM pathway was shown to function through the cell cycle-dependent element (CDE) and cell cycle genes homology region (CHR) promoter sites (19). The switch in protein binding to the CDE and CHR elements was suggested to control transcriptional downregulation by p53 upon activation of the p53-p21-DREAM-CDE/CHR pathway (19).

Genes regulated by CDE and CHR sites are categorized as *class I* and *class II* genes. Both classes contain functional CHR elements, while *class I* also employs a CDE four nucleotides upstream of a CHR (20). In contrast to CDE sites, CHR elements conform to a well-defined consensus (21). Many promoters of late cell cycle genes contain functional CHR elements, but do not require CDE sites (15,21). The requirement for CHR elements stems from the binding of the MuvB complex, which forms the core of DREAM and MMB, to CHR sites (15,21). Therefore, it is reasonable to categorize genes by their CHR (15,20,21). While bound by DREAM, they mediate transcriptional repression in G_0_ and G_1_. Later during the cell cycle, DREAM-mediated repression is lost and the CHR contributes to activation of the previously repressed genes by binding MuvB complexes which recruit B-MYB (MMB) and FOXM1 proteins (15,21,22).

Previously, three target genes regulated by the p53-p21-DREAM-CDE/CHR pathway were known, namely *CCNB2*, *KIF23* and *PLK4* (19,23,24). With the small number of established targets, the general importance of the p53-p21-DREAM-CDE/CHR pathway remained to be established. Thus, we took a genome-wide approach with the aim to identify possibly all genes targeted by this pathway.

The CHR elements of *CCNB2*, *KIF23* and *PLK4* display the canonical sequence TTTGAA or its functional inverse site TTCAAA (19,23,24). Here, we included a recent compilation of CHR sites deviating from the canonical TTTGAA (21). A total of ten CHR variants are known to date with examples represented by the cell cycle genes *Ccna2* (CHR sequence: CTTGAA) (25), *Ccnb1* (TTTAAA) (26), *Ccnb2* (TTTGAA) (15), *B-Myb* (*Mybl2*; TAGGAA) (27), *Bub1* (TTCGAA), *Chek2* (TTTGTA), *Melk* (TTTGAT), *Pold1* (TTTGAG), *Rad18* (TTCGAG) and *Rad54l* (TTCGAT) (21). Furthermore, we employed genome-wide chromatin immunoprecipitation (ChIP) data on DREAM binding (13) and genome-wide p53-dependent gene expression data from a meta-analysis (28). Our results provide a global map of 210 genes that are most likely regulated by the p53-p21-DREAM-CDE/CHR pathway. With many of these genes performing essential functions in G_2_ phase and mitosis, the results suggest that the p53-p21-DREAM-CDE/CHR pathway represents a key mechanism in p53-mediated cell cycle arrest.

## MATERIALS AND METHODS

### Computational analyses

Sources of primary data for the analyses were six studies on p53-dependent RNA expression (29–34) and ChIP results on binding of DREAM components E2F4, LIN9, LIN54 and p130 (13). A previously published list of 19 736 known protein-coding genes including their identifiers, p53 *Expression Score* and DREAM binding information served as a basis for the analyses (28). Furthermore, potential pathway target genes were selected for CHR sites annotated in the promoter regions of the genes. String representation for the CHR was chosen as follows: TTTGAA, TTTAAA, TTCGAA, TTTGTA, CTTGAA, TTTGAG, TTTGAT, TTCGAG, TTCGAT and TAGGAA (21). CHR elements were searched in the region of 1000 bp upstream and downstream from the transcriptional start site (TSS) on both strands that were not extended into the coding sequence or other genes located upstream of the TSS. PhastCons conservation scores (35) obtained from the multiz46 alignment of placental mammalia (36) were used to calculate average phylogenetic sequence conservation. Only those hits were listed that have an average PhastCons conservation score of at least 0.9. Results are displayed in Supplementary Table S2. Genes that are bound by DREAM, possess a conserved CHR and display a p53 *Expression Score* ≤ −3 are presented as strong candidate targets of the p53-p21-DREAM-CDE/CHR pathway in Table 1. Pathway enrichment analysis was carried out using the DAVID Functional Annotation tool (37).

**Table 1.** The p53-p21-DREAM-CDE/CHR signaling pathway regulates 210 potential target genes

ADSS	CDC25C	DLEU1	IFT80	MND1	RAD54L
ANLN	CDC7	DLGAP5	INCENP	MTF2	RANGAP1
ANP32E	CDCA2	ESCO2	ING1	MYBL2	RBM15
ARHGAP11A	CDCA3	ESPL1	ING3	NASP	RBMX
ARHGAP11B	CDCA5	EXOSC8	IQGAP3	NCAPD2	REEP4
ARL13B	CDCA8	EXOSC9	KIAA1731	NCAPD3	RIF1
ARL6IP1	CDK1	FAM64A	KIF11	NCAPG	RNASEH2A
ASF1B	CDK2	FAM83D	KIF14	NCAPG2	RTKN2
ASPM	CDKN3	FANCB	KIF15	NCAPH	SASS6
ATAD2	CENPA	FBXO5	KIF18A	NDC80	SCLT1
AURKA	CENPE	FOXM1	KIF20A	NEIL3	SGOL1
AURKB	CENPF	GAS2L3	KIF20B	NET1	SGOL2
BIRC5	CENPL	GSG2	KIF22	NUF2	SHCBP1
BORA	CENPM	GTSE1	KIF23	NUP107	SLC25A40
BUB1	CENPN	H2AFX	KIF24	NUP205	SMC2
BUB1B	CENPO	H2AFZ	KIF2C	NUP35	SMC4
C11orf82	CEP152	HAUS8	KIF4A	NUSAP1	SNRPA
C12orf32	CEP55	HIST1H2AE	KIFC1	OIP5	SP4
C15orf42	CHEK2	HIST1H2AM	KPNB1	ORC1	SPAG5
C2orf69	CIT	HIST1H2BF	LIN54	PCNT	SPC25
C3orf26	CKAP2L	HIST1H2BH	LIN9	PLK1	STIL
C9orf100	CKAP5	HIST1H2BI	LMNB1	PLK4	SUZ12
CACYBP	CKS1B	HIST1H2BM	LRRC49	POC5	TCERG1
CASC5	CKS2	HIST1H2BN	LSM5	POLD1	TMEM48
CBX3	CSE1L	HIST1H3C	MAD2L1	POLQ	TMPO
CCDC150	CSTF1	HIST1H3D	MASTL	POP7	TPX2
CCDC18	CTDSPL2	HIST1H4C	MCM5	PPIH	TRAIP
CCDC34	DARS2	HIST2H2AB	MCM7	PRC1	TROAP
CCDC99	DBF4B	HIST2H2AC	MCM8	PRIM2	TTK
CCNA2	DCAF16	HJURP	MDC1	PRPF38A	UBE2C
CCNB1	DCK	HMGB2	MELK	PRR11	UBE2S
CCNB2	DCLRE1B	HMMR	METTL13	PTTG1	UNG
CDC20	DDX10	HNRNPA0	METTL4	RACGAP1	USP1
CDC25A	DEPDC1	HNRNPA2B1	MIS18BP1	RAD18	YEATS4
CDC25B	DEPDC1B	HNRNPUL1	MKI67	RAD21	ZNF367

### Cell culture and drug treatment

HCT116 wild-type and HCT116 *p21*^−/−^ cells (38), HFF, and NIH3T3 cells (DSMZ, Braunschweig, Germany) were grown in Dulbecco's modified Eagle's medium (DMEM; Lonza, Basel, Switzerland) supplemented with 10% fetal calf serum (FCS) (Biochrom, Berlin, Germany) and penicillin/streptomycin and maintained at 37°C and 10% CO_2_. Cells were treated with DMSO (15 μl), doxorubicin (0.2 μg/ml; Medac, Wedel, Germany), nutlin-3a (10 μM; Cayman Chemicals, Ann Arbor, MI, USA) or 5-FU (25 μg/ml; Sigma, Taufkirchen, Germany) as indicated.

### Flow cytometry

Cells were fixed for at least 12 h at 4°C in one volume phosphate-buffered saline/1 mM EDTA and three volumes of absolute ethanol. DNA was stained with propidium iodide at a final concentration of 10 μg/ml in presence of RNase A (10 μg/ml). DNA content per cell was measured by flow cytometry on an LSR II instrument (Becton Dickinson, Franklin Lakes, NJ, USA). Cell sorting was carried out on a FACSVantage SE (Becton Dickinson). Data analysis was carried out with WinMDI 2.9 software.

### RNA extraction, reverse transcription and semi-quantitative real-time polymerase chain reaction (PCR)

Total cellular RNA was isolated using TRIzol Reagent (Invitrogen, Carlsbad, CA, USA) following the manufacturer's protocol. One-step reverse transcription and quantitative real-time PCR were performed with an ABI 7300 Real-Time PCR System (Applied Biosystems, Forster City, CA, USA) using QuantiTect SYBRGreen PCR Kit (Qiagen, Hilden, Germany) as described previously (19,24). Primer sequences are listed in Supplementary Table S1.

### Chromatin immunoprecipitation (ChIP)

HCT116 and HFF cells were cross-linked with 1% formaldehyde for 10 min at room temperature. ChIP was performed as described previously (19,24). The following antibodies were used for precipitation of transcription factors: E2F4 (C-20, Santa Cruz Biotech.), p130 (C-20, Santa Cruz Biotech.), p53 (Ab-6, DO-1, Calbiochem), LIN9 (ab62329, Abcam, Cambridge, UK). A second LIN9 antibody was a kind gift from James DeCaprio (13). A non-targeting IgG polyclonal rabbit antibody was used as a control for non-specific signals. For all precipitations 1–2 μg of antibody and 20–35 μl of Protein G Dynabead suspension (Invitrogen) were used. Immunoprecipitated DNA was used as template for quantitative real-time PCR as described previously (19,24). Primer sequences are listed in Supplementary Table S1.

## RESULTS

### The p53-p21-DREAM-CDE/CHR signaling pathway regulates 210 potential target genes

Three sets of information were utilized to identify possibly all genes regulated by the p53-p21-DREAM-CDE/CHR pathway: Genome-wide RNA expression dependent on p53, ChIP data on binding of DREAM components in the human genome, and genome-wide detection of phylogenetically conserved CHR elements.

Data from a meta-analysis of six genome-wide p53-dependent gene expression analyses to identify genes that are repressed by p53 formed one basis for the analysis (28–34). In each study, a gene could be identified as activated (positive score; +1) or repressed (negative score; −1) by p53. By calculating the sum over all analyses, p53-dependent *Expression Scores* ranging from −6 to +6 were assigned, representing direction as well as reproducibility of regulation (28). An *Expression Score* ≤ −3 was a criterion for a gene to be considered as repressed by p53. As a second data set, binding results of DREAM components E2F4, LIN9, LIN54 and p130 from ChIP analyses were employed to detect DREAM target genes (13). A gene was scored positive for DREAM binding if three of the four components were detected. The third selection criterion was a conserved CHR site in the promoter of a gene. CDE sites were not used because many potential target genes were expected to employ solely a CHR and not require a CDE (20). Since all CDE sites require CHR elements for their function, focusing the search on CHR sites covers all CHR- and CDE/CHR-regulated genes (20,21). *CCNB2*, *KIF23* and *PLK4*, the three genes thus far shown to be regulated by the p53-p21-DREAM-CDE/CHR pathway, bind DREAM through the canonical CHR sequence TTTGAA (19,23,24). Recently, variants of CHR sites were identified in a comprehensive genome-wide screen. This yielded a list of ten functional CHR variants, namely TTTGAA, TTTAAA, TTCGAA, TTTGTA, CTTGAA, TTTGAG, TTTGAT, TTCGAG, TTCGAT and TAGGAA (21). Here, we employed all of these CHR motifs in a genome-wide search to obtain an essentially complete set of genes regulated via CHR- and CDE/CHR sites. In order to score positive as a CHR site, phylogenetic conservation of potential CHRs within 1000 bp upstream or downstream of annotated transcriptional start sites (TSS) was required.

**Figure 1.**
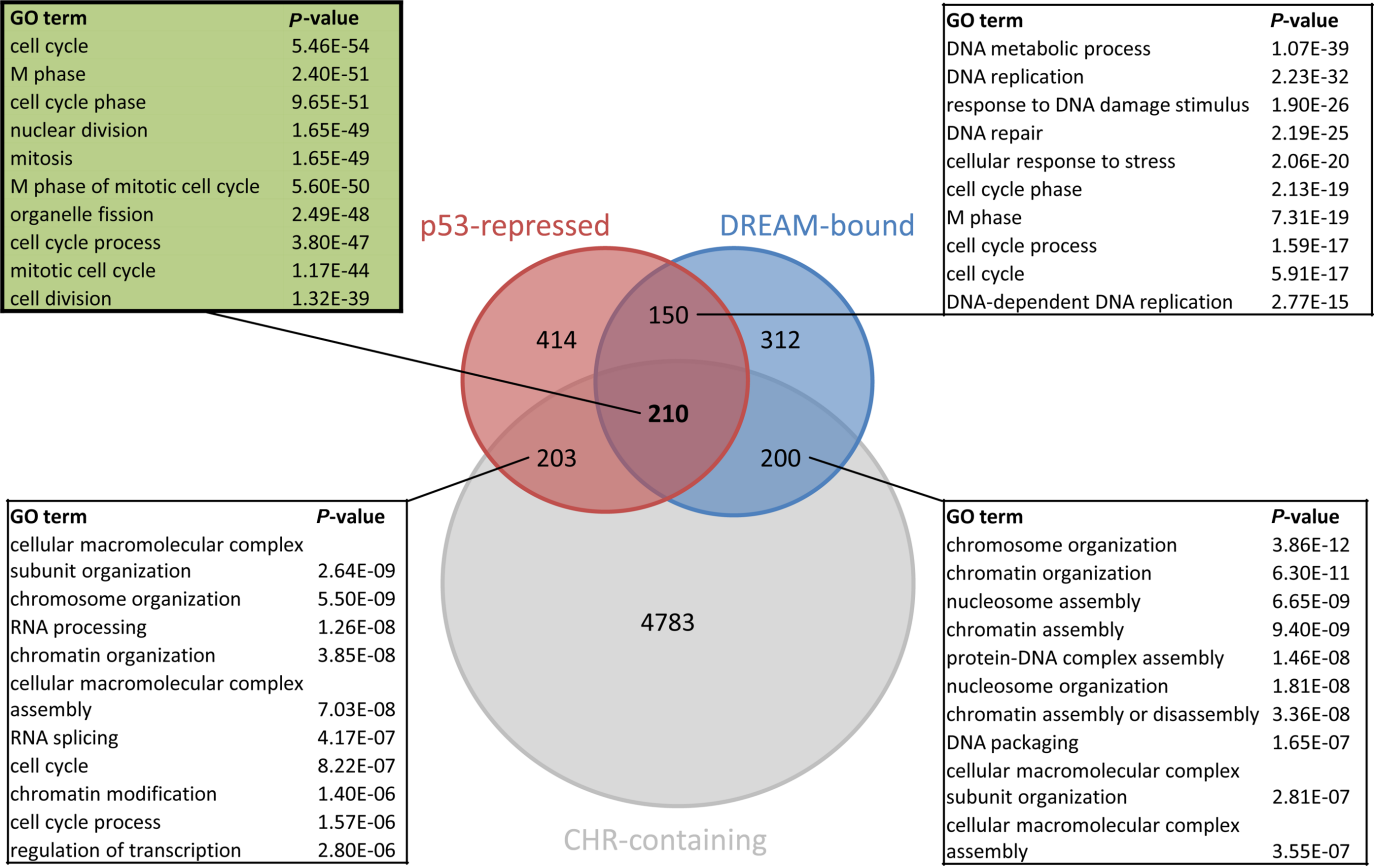
Genes associated with cell cycle and mitosis are enriched among targets of the p53-p21-DREAM-CDE/CHR pathway. Venn diagram displaying the overlap in the groups of genes found as repressed by p53, bound by DREAM, and containing a conserved CHR element. Top 10 GO terms enriched among the 210 genes displayed in Table 1 and the other group overlaps as identified using the DAVID Functional Annotation tool (Supplementary Table S3).

We found 870 genes bound by DREAM in proximity to their TSS (Supplementary Table S2). Out of these 870 genes, 358 (41.1%) were repressed by p53 displaying an *Expression Score* of ≤ −3. When all three selection criteria were applied with binding of three out of four DREAM components, repression by p53 with an *Expression Score* ≤ −3, and presence of one of the ten CHR variants being phylogenetically conserved in the promoter, we identified 210 genes as targets for the p53-p21-DREAM-CDE/CHR pathway (Figure 1; Table 1, Supplementary Table S2). The three known targets of the pathway, *CCNB2*, *KIF23* and *PLK4*, were detected by the analysis. This demonstrates the ability of this screening approach to identify *bona fide* candidates. *GAS2L3* had been missed by the search because only two of the DREAM components were above the threshold (Supplementary Table S2), although it had been described as a DREAM target (39). Also, *B-MYB (MYBL2)* was previously shown to be a CHR-controlled gene downregulated by p53 (21,28). Therefore, *GAS2L3* and *MYBL2* were included in the list of genes regulated by the p53-p21-DREAM-CDE/CHR pathway (Table 1).

### Repression of non-canonical CHR-containing genes by p53 requires p21 and is conserved between mouse and human

**Figure 2.**
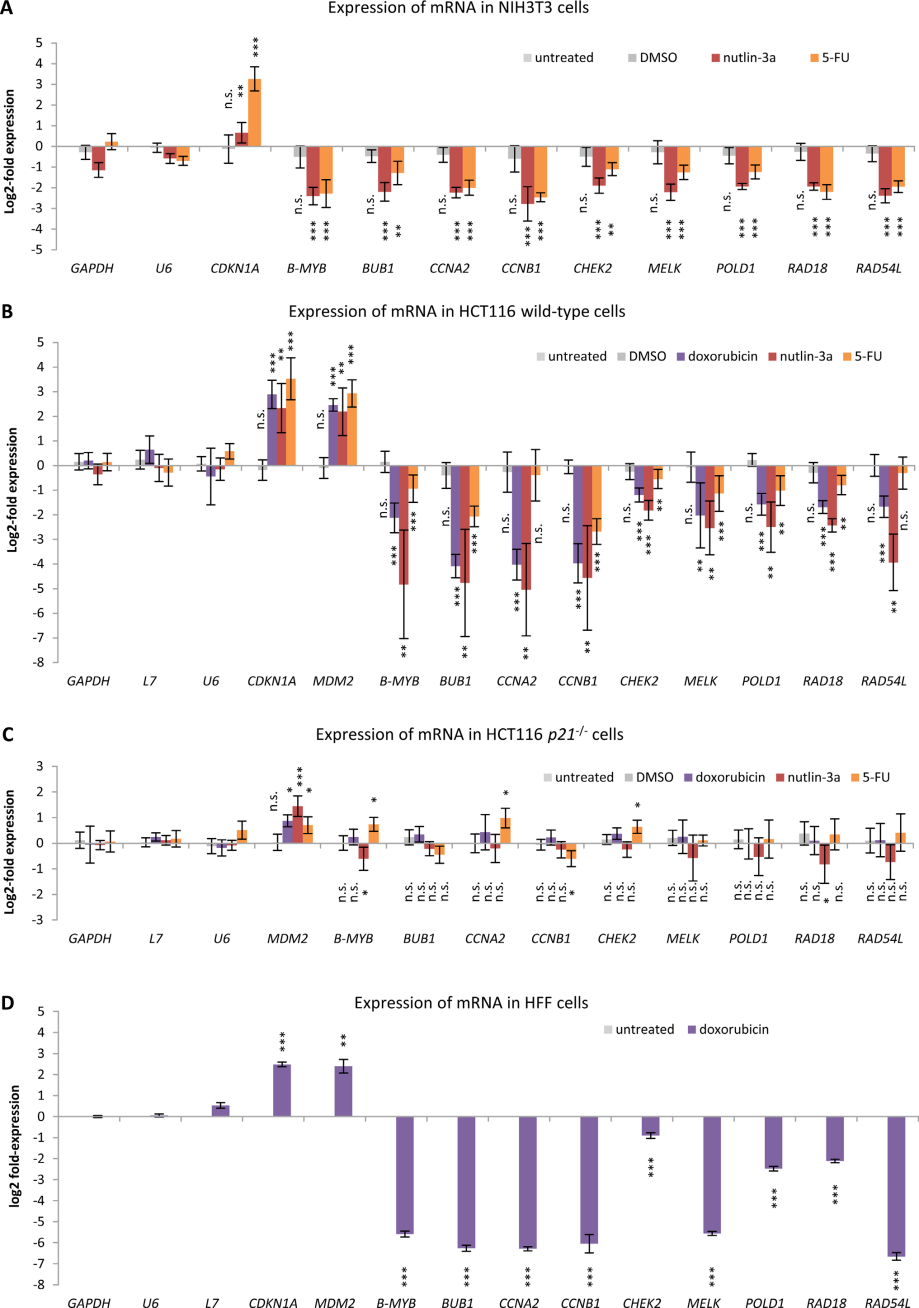
Repression of non-canonical CHR-containing genes by p53 requires p21 and is conserved between mouse and human. The log_2_-fold change of mRNA expression from treated compared to untreated (**A**) NIH3T3, (**B**) HCT116 wild-type, (**C**) HCT116 *p21*^−/−^ and (**D**) HFF cells is displayed. Cells were treated with doxorubicin, nutlin-3a or 5-FU for 24 h. Untreated cells and cells treated with DMSO served as controls. Normalization was carried out against measurements from untreated cells. *GAPDH*, *L7* and *U6* served as negative controls for p53 response, while *CDKN1A* and *MDM2* were employed as positive controls. (A–C) Experiments were performed with two biological replicates and three technical replicates each (*n* = 6). (D) Experiments were performed with three technical replicates (*n* = 3). Significance of changes in expression levels was tested against *GAPDH* expression levels using the unpaired Student's *t*-test; n.s. not significant; *P ≤ 0.05; **P ≤ 0.01; ***P ≤ 0.001.

With *CCNB2*, *KIF23* and *PLK4*, only genes containing canonical CHR sites had been tested in detail for their regulation through the p53-p21-DREAM-CDE/CHR pathway. In order to examine several of the new targets identified through the bioinformatic analysis for their regulation on a single-gene basis, we chose candidate genes which mostly contain CHR sites deviating from the canonical TTTGAA. We investigated p53-dependent regulation of *B-MYB* (*MYBL2*), *BUB1*, *CCNA2*, *CCNB1*, *CHEK2*, *MELK*, *POLD1*, *RAD18* and *RAD54L*, which hold representative non-canonical CHR motifs that have been shown to bind DREAM (21). Relative mRNA expression was determined in untreated NIH3T3 cells in comparison to cells treated with the solvent DMSO, the MDM2 inhibitor nutlin-3a or the pyrimidine analogue 5-FU. We used NIH3T3 cells because cell cycle-dependent regulation of these genes was demonstrated in this cell line in a previous study (21) and results from this mouse cell line would complement the data from the human cell systems (Supplementary Table S2). Indeed, we found all genes with non-canonical CHR elements to be downregulated upon p53 activation (Figure 2A, Supplementary Figure S1). Next, we investigated p53-dependent regulation of the human gene orthologs in HCT116 wild-type, HCT116 *p21*-negative and non-cancerous HFF cells (Figure 2B–D, Supplementary Figure S1). Again, we found all genes containing a non-canonical CHR to be repressed following p53 activation in HCT116 and HFF cells, similar to the observations in NIH3T3 cells. These results support the data from our genome-wide screening and provide evidence that p53-dependent repression of these genes is conserved between mouse and human. Importantly, p53-dependent repression is essentially lost in HCT116 *p21*^−/−^ cells (Figure 2C). In agreement with our data, p21-dependent downregulation has been reported for four of the nine genes tested here, namely *CCNA2* (4,6,11,40), *CCNB1* (6–8,11), *POLD1* (11) and *RAD54L* (7). These results are consistent with a mechanism involving indirect, p21-mediated repression upon p53 stabilization. Based on these findings, we conclude that p53-dependent repression of these CHR-containing genes requires p21.

### p53 does not bind to promoters of genes harboring CHR sites

**Figure 3.**
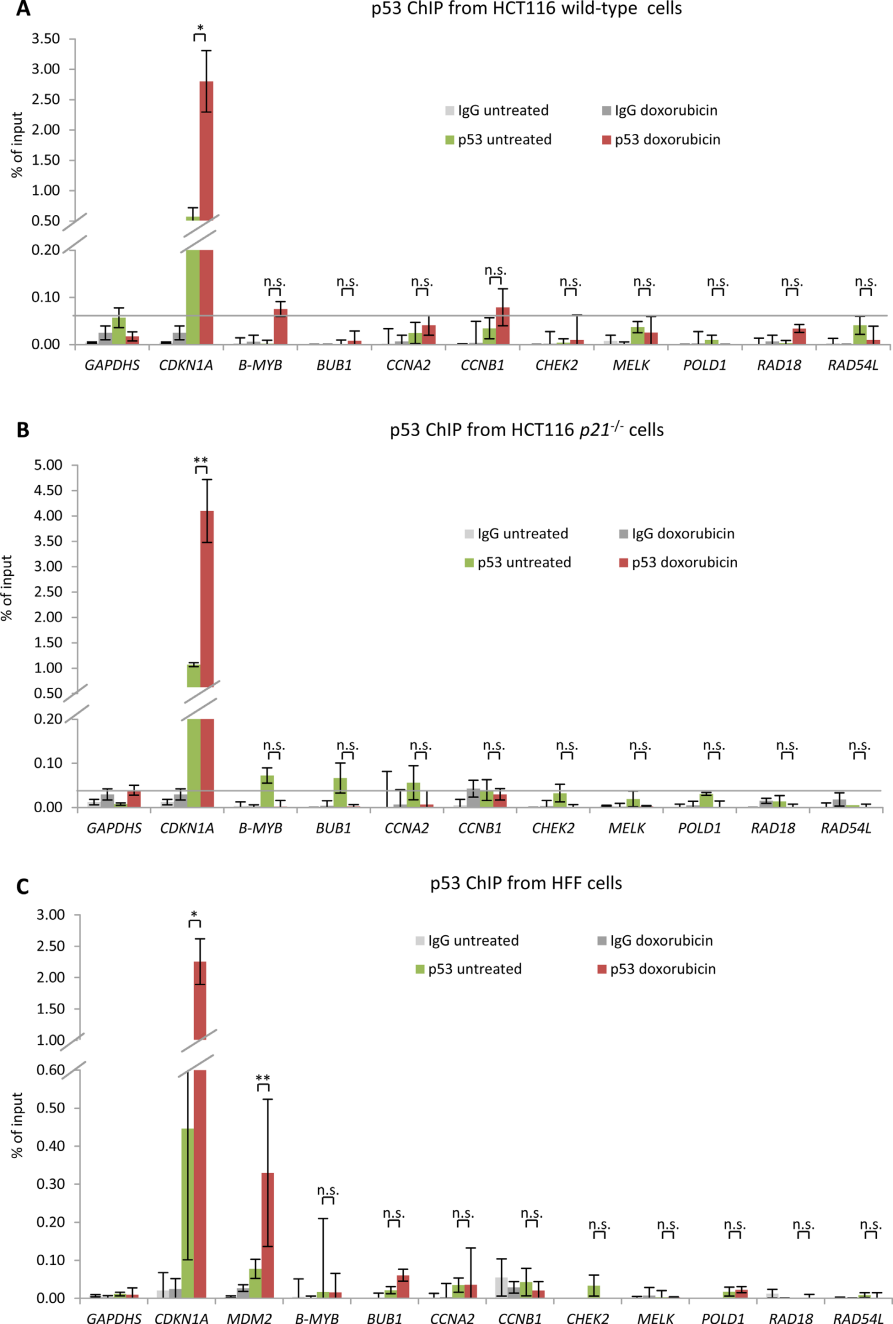
p53 does not bind to promoters of genes harboring CHR sites. Protein binding to promoters of the indicated genes was tested by ChIP in **(A)** HCT116 wild-type cells, **(B)** HCT116 *p21*^−/−^ and **(C)** HFF cells either left untreated or treated with doxorubicin for 24 h (HFF) or 48 h (HCT116) followed by real-time PCR. The *CDKN1A* promoter served as a positive control for p53 binding; the *GAPDHS* promoter which does not bind p53 was used as a negative control. One representative experiment with three technical replicates (*n* = 3) is displayed.

The finding that all genes tested require p21 for p53-dependent repression contrasts the previously proposed direct repression by p53 for two of the genes, *CCNB1* and *POLD1* (41–44). Thus, we tested for p53 binding to the promoters of *CCNB1* and *POLD1* as well as *B-MYB* (*MYBL2*), *BUB1*, *CCNA2*, *CHEK2*, *MELK*, *RAD18* and *RAD54L* in HCT116 wild-type, HCT116 *p21*^−/−^, or HFF cells, untreated or treated with doxorubicin (Figure 3). Binding of p53 to promoters with CHR sites was not observed. These findings do not support a mechanism of direct repression by p53 (41–44), but are consistent with the indirect p53-p21-DREAM-CDE/CHR pathway which requires p21 (Figure 2).

### p53 induces p21-dependent binding of DREAM to genes with CHR sites

**Figure 4.**
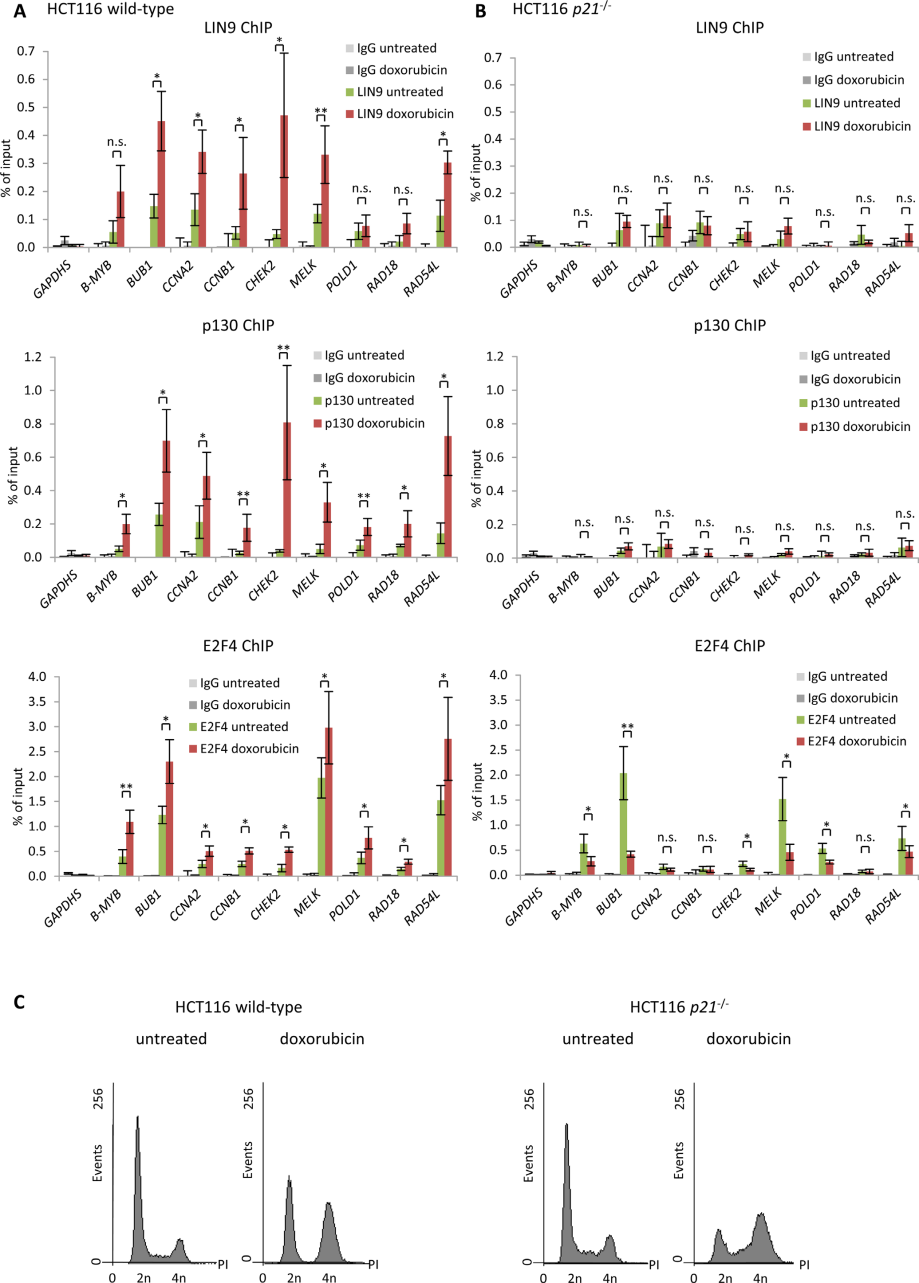
p53 induces p21-dependent binding of DREAM to genes with CHR sites. Protein binding to CHR-containing promoters in untreated or 48 h doxorubicin-treated HCT116 **(A)** wild-type or **(B)** *p21*^−/−^ cells was tested by ChIP followed by real-time PCR. Protein binding to the *GAPDHS* promoter served as negative control. One representative experiment with three technical replicates (*n* = 3) is displayed. Significance was tested using the paired Student's *t*-test; n.s. not significant; *P ≤ 0.05; **P ≤ 0.01; ***P ≤ 0.001. **(C)** Flow cytometry of cells used in Figure 4; cells were stained with propidium iodide (PI).

The CHR sites in the *B-MYB* (*MYBL2*), *BUB1*, *CCNA2*, *CCNB1*, *CHEK2*, *MELK*, *POLD1*, *RAD18* and *RAD54L* genes have previously been shown by DNA affinity purification assays to bind DREAM *in vitro* (21). Here, we tested for differential DREAM binding by ChIP in cells before and after p53 activation. Binding of the DREAM components LIN9, E2F4 and p130 to the promoters of these genes were examined upon p53 induction by doxorubicin. Binding of the MuvB core component LIN9 was found to be enriched at some but not all promoters (Figure 4A). In contrast, the DREAM components E2F4 and p130 showed increased binding at all CHR-containing promoters in HCT116 wild-type cells treated with doxorubicin compared to untreated cells (Figure 4A). These findings are consistent with the model developed from the regulation of *CCNB2*, *KIF23* and *PLK4*, which contain canonical CHR sites (19,23,24). Moreover, DREAM binding to the promoters was generally reduced in HCT116 *p21*^−/−^ cells and did not increase after doxorubicin treatment of HCT116 *p21*^−/−^ cells (Figure 4B). Interestingly, binding of E2F4 was observed for most genes to be reduced in doxorubicin-treated compared to untreated HCT116 *p21*^−/−^ cells (Figure 4B). A likely explanation is the cell cycle shift toward G_2_/M upon doxorubicin treatment that causes the expected decrease in DREAM stability when p21 cannot inhibit the CDKs (Figure 4B and C). Thus, the p53-p21 pathway appears to stabilize formation of DREAM and enhances its binding to promoters in wild-type cells despite the doxorubicin-induced G_2_/M cell cycle shift. Taken together, the observations on these nine individual genes suggest that p21 is required for DREAM binding to CHR promoters upon p53 activation.

### G_2_/M cell cycle genes are among the targets of the p53-p21-DREAM-CDE/CHR signaling pathway

We performed a Gene Ontology analysis of the 210 target genes using the DAVID Functional Annotation tool (37). Cell cycle-associated terms were prominently represented in this set of genes (Figure 1, Supplementary Table S3). Particularly, genes encoding proteins required in late phases of the cell cycle from G_2_ through mitosis comprised the largest fraction in the analysis. When comparing the 210 genes to an analysis of four genome-wide cell cycle expression studies (21), it was observed that 158 (75.2%) of the CHR genes show cell cycle-dependent regulation. More precisely, the vast majority of genes regulated by the p53-p21-DREAM-CDE/CHR pathway displayed their peak expression in G_2_ phase or mitosis.

## DISCUSSION

In this study, a list of target genes for the p53-p21-DREAM-CDE/CHR signaling pathway is presented. Selection of the 210 candidate targets was performed using stringent criteria to minimize the number of false-positives. Identification of the known p53-p21-DREAM-CDE/CHR targets *CCNB2*, *KIF23* and *PLK4* demonstrates the ability of the screening approach to identify *bona fide* candidates. These three genes harbor the canonical CHR sequence TTTGAA in their promoters (19,23,24). Notably, the non-canonical CHR elements TTTAAA, TTCGAA, TTTGTA, CTTGAA, TTTGAG, TTTGAT, TTCGAG, TTCGAT and TAGGAA had not yet been tested for their function in the p53-p21-DREAM-CDE/CHR pathway (21). Thus, *B-MYB* (*MYBL2*), *BUB1*, *CCNA2*, *CCNB1*, *CHEK2*, *MELK*, *POLD1*, *RAD18* and *RAD54L* as representative genes harboring the nine non-canonical CHR variants were tested for their response to p53 activation. Detailed experiments with all of these genes confirmed results from the genome-wide analyses and supported the notion that the genes are regulated by this pathway (Figures 2–4). DREAM, represented by its components p130 and E2F4, was shown to be recruited to their promoters (Figure 4) (19). DREAM binding to the specific CHR sites has also been established (21). In agreement with our observations, it has been reported that p53-dependent downregulation of the mouse homologs *Bub1*, *Ccna2* and *Ccnb1* requires p107 and p130 (45). These pRB-related pocket proteins were later identified as components of the DREAM complex (13,14,18). This leads to the conclusion that the p53-p21-DREAM-CDE/CHR pathway functions through all known CHR variants and is largely conserved between mouse and human.

Furthermore, there are two indicators that threshold settings for the genome-wide analysis were adjusted to yield high-confidence candidates. One indicator is that all candidates tested in detail for their regulation by the pathway were confirmed (Figures 2–4). This suggests that the rate of false-positives may be low. The other observation is that *GAS2L3* was not selected as a pathway gene. Since the DREAM binding threshold required three of the four DREAM components tested to be detected in the ChIP screen. However, in the case of *GAS2L3* only two components were found positive for binding in the screen (Supplementary Table S2) (13), although *GAS2L3* had been reported to be a DREAM target (39). Taken together, these observations indicate that threshold settings were so stringent that the computational analysis rather missed candidates than to include false-positive genes. This suggests that the 210 genes in Table 1 are indeed strong candidate targets of the p53-p21-DREAM-CDE/CHR pathway but that some additional target genes may have been missed.

GO term enrichment analysis of the 203 genes that contain a conserved CHR and are repressed by p53, but do not bind DREAM, shows a small enrichment also for cell cycle genes (Figure 1). Considering that *GAS2L3* belonged to that group before we manually assigned it as DREAM target, it is likely that more currently unknown DREAM target genes can be found among this group of genes. The group of 200 genes binding DREAM, possessing a conserved CHR, and that are not found to be repressed by p53 likely contains genes that either are repressed by p53 but were missed due to the high stringency criteria in our analysis or genes that might be regulated solely by DREAM in the cell cycle. Interestingly, GO term analysis of the 150 genes that bind DREAM and are repressed by p53, but do not possess phylogenetically conserved CHR elements, shows high enrichment for biological processes of early (G_1_/S) cell cycle genes, such as DNA replication and DNA metabolism (Figure 1, Supplementary Table S3).

Cell cycle genes are general targets for p53-mediated downregulation independent of cell type or method of p53 activation. In regard to the underlying mechanism, either no or contradictory details have been reported. Several cell cycle genes among the 210 targets have been suggested to be directly repressed by p53. Examples include *ANLN* (46), *AURKA* (29), *CDC20* (47), *CDC25B* (48), *CDK1* (*CDC2*) (49), *PRC1* (50) or *PTTG1* (51). However, these genes were not found to be directly repressed by p53 in several genome-wide studies (52–54). Moreover, recent evidence suggests that p53 does not directly repress target genes (28). These observations are consistent with our data, which provide no evidence for direct repression of *CCNB1* and *POLD1*, although these genes had been reported as direct p53 targets (Figures 2–4) (41–44). Additionally, for a large number of cell cycle genes the mechanism underlying p53-dependent repression remained unresolved, e. g. *CDC25A* (55), *CKS1B* (56), *CKS2* (57) or *HMMR* (*RHAMM*) (58). However, for several other genes indirect repression via p21 was reported, including *BUB1B* (59,60), *CENPA* (61), *CENPE* (61), *CENPF* (59), *FOXM1* (62) and *MAD2L1* (59,60). All of these genes can be found among the 210 target genes (Table 1). Two previous studies had suggested that many cell cycle genes are repressed by p53 via p21 and E2F4, with the earlier of the two reports showing DREAM components and CHR sites as being part of this regulation and the later comparing E2F4 binding with p53-dependent expression data (19,63). Here, we took a genome-wide approach combining p53-dependent expression, binding of several DREAM components and selection of CHR-containing genes. We provide evidence that the E2F4-containing DREAM complex represses many, if not all, of these genes upon p53 activation and that this complex is targeted to many promoters via CDE/CHR motifs. Thus, the present analysis offers a mechanism for p53-dependent repression of these genes, resolving contradictions between earlier reports.

Interestingly, expression of most of the 210 candidate genes varies during the cell cycle. Expression levels peak in the late cell cycle phases, which correspond to the function of their encoded proteins in G_2_ and mitosis. Thus, the results suggest that repression of these cell cycle genes serves as a mechanism for p53 to stop cell division, particularly in G_2_/M. This mechanism of p53-dependent cell cycle control is exemplified by downregulation of key cell cycle regulators such as the cyclins *CCNA2, CCNB1* and *CCNB2*, the cyclin-dependent kinases *CDK1* and *CDK2*, as well as the kinases and phosphatases *AURKA*, *AURKB*, *PLK1*, *PLK4*, *CHEK2*, *CDC25A* and *CDC25C*. In addition to fast responses which stop cell cycle progression such as transcriptional suppression by *cyclin F* (64), the p53-p21-DREAM-CDE/CHR pathway may contribute to a permanent cell cycle arrest. In agreement with this model, the DREAM complex was shown to be important for permanent proliferation arrest during senescence (65).

Taken together, we establish a target list of the p53-p21-DREAM-CDE/CHR signaling pathway. The results also suggest a mechanism for the regulation of cell cycle genes that are targeted by this pathway. Most of the genes are dominantly expressed in G_2_ phase and mitosis and essential for the progression through the late cell cycle. Thus, downregulation of these genes through the p53-p21-DREAM-CDE/CHR pathway appears to be a principal mechanism for G_2_/M cell cycle arrest by p53.

## Supplementary Material

SUPPLEMENTARY DATA
