# Supplementary material for: The p53-p21-DREAM-CDE/CHR pathway regulates G2/M cell cycle genes
Source: Nucleic Acids Res. 2015 Sep 17;44(1):164–74. doi: 10.1093/nar/gkv927 (PMC4705690; doi:10.1093/nar/gkv927)
Supplement: SUPPLEMENTARY DATA [file supp_44_1_164__index.html]

The p53-p21-DREAM-CDE/CHR pathway regulates G2/M cell cycle genes — SUPPLEMENTARY DATA 

# The p53-p21-DREAM-CDE/CHR pathway regulates G2/M cell cycle genes

## SUPPLEMENTARY DATA

- SUPPLEMENTARY DATA
- SUPPLEMENTARY DATA
- SUPPLEMENTARY DATA
- SUPPLEMENTARY DATA
