# Supplementary material for: The p53-p21-DREAM-CDE/CHR pathway regulates G2/M cell cycle genes
Source: Nucleic Acids Res. 2015 Sep 17;44(1):164–74. doi: 10.1093/nar/gkv927 (PMC4705690; doi:10.1093/nar/gkv927)
Supplement: SUPPLEMENTARY DATA [file supp_gkv927_nar-01392-x-2015-File004.docx]

**Legends for Supplementary Material**

**Supplementary Figure S1**

Flow cytometry of cells used in Figure 1; cells were stained with propidium iodide (PI).

**Supplementary Table S1**

Primer sequences used for real-time PCR (qPCR) measurements.

**Supplementary Table S2**

Collated data on 19,736 protein-coding genes including their identifiers, p53 *Expression Score*, DREAM binding, and presence of phylogenetically conserved (PhastCons score ≥ 0.9) CHR elements within +/- 1000 bp from their transcription start site (TSS).

**Supplementary Table S3**

GO enrichment analysis of the 210 target genes of the p53-p21-DREAM-CDE/CHR pathway was performed using the DAVID Functional Annotation tool.
